# Nephrotoxicity and ototoxic symptoms of injectable second-line anti-tubercular drugs among patients treated for MDR-TB in Ethiopia: a retrospective cohort study

**DOI:** 10.1186/s40360-019-0313-y

**Published:** 2019-05-23

**Authors:** Workineh Shibeshi, Anandi N. Sheth, Addisu Admasu, Alemseged Beyene Berha, Zenebe Negash, Getnet Yimer

**Affiliations:** 1 Department of Pharmacology & Clinical Pharmacy, School of Pharmacy, College of Health Sciences, Addis Ababa University, Addis Ababa, Ethiopia; 2 Division of Infectious Diseases, Department of Medicine, Emory University School of Medicine, Atlanta, GA USA; 3 St.Peter’s Specialized Hospital, Addis Ababa, Ethiopia; 4 Global One Health initiative, Eastern Africa Regional Office, Office of International Affairs, The Ohio State University, Addis Ababa, Ethiopia

**Keywords:** Nephrotoxicity, Ototoxicity, MDR-TB, Injectables

## Abstract

**Background:**

Nephrotoxicity and ototoxicity are clinically significant dose-related adverse effects associated with second-line anti-tubercular injectables drugs (aminoglycosides and capreomycin) used during intensive phase of treatment of multi-drug resistant tuberculosis (MDR-TB) patients. Data are scarce on injectable-induced nephrotoxicity and ototoxicity in Ethiopian MDR-TB patients. The aim of this study was to assess the prevalence, management of nephrotoxicity and ototoxic symptoms and treatment outcomes of patients treated for MDR-TB with injectable-based regimens.

**Method:**

This was retrospective cohort study based on review of medical records of about 900 patients on MDR-TB treatment from January 2010 to December 2015 at two large TB referral hospitals in Addis Ababa, Ethiopia. Nephrotoxicity in study participants was screened using baseline and monthly measurement of serum creatinine and clinical diagnosis and patient reports.

**Results:**

Overall, 473 (54.2%) of participants were male. Children accounted for 47 (5.5%) of cases and the mean age of participants was 32 ± 12.6 years with range of 2–75 years. The majority (*n* = 788, 84.6%) of participants had past history of TB. The most commonly used injectable anti-TB drug was capreomycin (*n* = 789, 84.7%), while kanamycin and amikacin were also used. There was a statistically significant increment (p<0.05) in the mean serum creatinine values from baseline throughout intensive phase of treatment with a 10–18% prevalence of nephrotoxicity. Based on clinical criteria, nephrotoxicity was detected in 62 (6.7%) and ototoxic symptoms were detected in 42 (4.8%) participants. Nephrotoxicity and ototoxic symptoms were clinically managed by modification of treatment regimens including dose and frequency of drug administration.

**Conclusion:**

Nephrotoxicity and ototoxic symptoms were significant problems among patients on follow-up for MDR-TB treatment. Based on laboratory criteria (serum creatinine), nephrotoxicity remained significant adverse events throughout intensive phase of treatment, indicating close monitoring of patients for successful outcome is mandatory until countries adopt the recent injectable-free WHO guideline and under specific conditions.

## Background

Multidrug-resistant tuberculosis (MDR-TB) is caused by strains of *M.tuberculosis* that are resistant to at least isoniazid and rifampicin, and is an important clinical and public health threat worldwide [1]. According to the WHO Global tuberculosis report 2018, Ethiopia stands 7th among 20 high TB-burden countries [2]. According to this report, in 2017 estimated incidence of multidrug-resistant tuberculosis or rifampicin-resistant tuberculosis (MDR/RR-TB) per 100, 000 population was 5.2 (2.8–8.4). A systematic review of the literature on MDR-TB in Ethiopia indicates that among the 23 studies conducted, six of them reported high prevalence of MDR-TB in the range of 3.3–46.3% [3].

There are a number of currently available WHO-recommended diagnostic techniques for detection of resistance of *M. tuberculosis* including phenotypic assays, and genotypic methods such as GeneXpert MTB/RIF assay and Line probe assay (LPA) that detect specific DNA mutations in the genome of the *M. tuberculosis* associated with resistance to specific anti-TB drugs [4].

The treatment of MDR-TB requires long duration (18–24 months) of administration of second-line anti-TB drugs. In WHO 2016 guideline [5] a shorter MDR-TB treatment regimen was recommended under specific conditions, and the drugs were regrouped into five categories that recommends for RR-TB or MDR-TB, a regimen with at least five effective TB medicines during the intensive phase of treatment. However, WHO 2018 guideline [6] regrouped medicines into three categories based on the latest evidence about the balance of effectiveness to safety. The preferred options for treatment now includes a fully oral injectable-sparing regimen. Kanamycin and capreomycin are not recommended in WHO 2018 guideline as they were associated with treatment failure and relapse [6].

Second-line injectable agents including aminoglycosides (amikacin, kanamycin, streptomycin), and mechanistically related polypeptide drug (capreomycin) in combination with fluoroquinolones, form the backbone for the treatment of multidrug-resistant tuberculosis, and also recommended by the earlier WHO guidelines [7]. A crucial issue related to long-term administration of the injectable group is its toxicity which affects adherence and treatment outcomes [8]. Nephrotoxicity and ototoxicity and are well known dose-related adverse effects of aminoglycosides and major concerns because of the narrow therapeutic range of these agents and the wide variability in pharmacokinetics among patients [9].

Aminoglycosides selectively concentrate in the cochlea and vestibular system and are taken up into lysosomes and mitochondria in hair cells causing permanent hearing loss by damaging inner ear sensory cells. Ototoxicity refers to damage of inner ear structures (cochlea and vestibule) and their function (hearing and balance) following exposure to specific medications [10]. Vestibulotoxicity occurs in up to 15% of patients after aminoglycoside administration, whereas cochleotoxicity occurs in 2 to 25% of patients [11]. Although adverse events are poorly reported, about 7.3% of adult patients and 10.1% children had serious adverse events attributed to second-line injectable agents [5]. In a study focused on hearing loss in children with tuberculosis, 24% of children treated for MDR-TB with a regimen containing injectable agent had hearing loss and 64% of children had progression of hearing loss after completion of treatment [12].

The mechanisms of aminoglycoside-induced toxicity generally includes drug trafficking across endothelial and epithelial barrier layers, sensory cell uptake of drugs and disruption of intracellular physiological pathways [13]. The molecular mechanisms of aminoglycoside-induced ototoxicity are not clearly elucidated, but in some subjects aminoglycoside-induced ototoxicity is associated with A1555G polymorphism in 12S rRNA that results in defective protein synthesis which is a primary mechanism of cytotoxicity [13]. Other mitochondrial 12S rRNA mutations, including C1494T and T1095C, also increase susceptibility to aminoglycoside ototoxicity. Aminoglycosides also inhibit the activity of antioxidant enzymes and induce overproduction of reactive oxygen species in cochlear cells [14, 15].

Aminoglycoside-induced nephrotoxicity is due to their selective accumulation in the proximal renal tubules that preferentially takes up aminoglycosides compared to more distal tubular regions. Aminoglycosides are freely filtered across the glomerulus with 5 to 10% of a parenteral dose being taken up and sequestered by the proximal tubule cells where the aminoglycoside can achieve concentrations vastly exceeding the concurrent serum concentration [16]. The proximal cellular uptake of aminoglycosides is effected by binding of aminoglycoside to megalin-cubulin complex that facilitates its endocytosis [13].

Current evidence indicates that second-line injectable agents are associated with an increased likelihood of treatment success when included in a conventional MDR-TB treatment regimen. It was recommended that adults with RR- /MDR-TB always receive a second-line injectable agent as part of their regimen unless there is an important contraindication [5], however, due to their serious adverse events, WHO 2018 Guideline has an option for injectable-free regimens [6]. In resource-limited settings, the consequences of adverse drug reactions (ADRs) is alarming because the health facilities and specialist services required for management are scarce; hence, long term administration of second-line anti-TB drugs may result in permanent disability, increased morbidity, mortality, losses to follow-up, increased health care costs, treatment interruptions, treatment failure and as a consequence, the emergence of further drug resistance [17, 18]. As a result, WHO has recommended identification and early aggressive management of adverse drug reactions as an essential component of the care of MDR-TB patients which increases adherence to treatment as key to successful outcome [19].

Ethiopia initiated the first MDR-TB treatment in 2009 at St. Peter’s Specialized Hospital in Addis Ababa [20], and there has been a rapid scale-up of drug resistant TB care in recent years at the national and regional state level; and currently there are over 46 care sites for drug-resistant tuberculosis. In Ethiopia, specific studies on two major adverse drug reactions (ototoxicity and nephrotoxicity) associated with use of injectable second-line drugs in patients treated for MDR-TB are scarce. Previous study [21] has reported the prevalence and risk factors of all adverse drug reactions associated with MDR-TB treatment in two treatment centers in Addis Ababa. This study was a cross-sectional study which used small number (*n* = 72) patients and children were excluded despite their high risk for toxicity [12]. In previous study [21], hearing loss was reported in 6 patients (8.3%), and renal failure was reported in only 5 patients (6.9%); however, this study focused on description of all adverse reactions associated with all regimens of MDR-TB, and did not undertake specific study of ototoxicity and nephrotoxicity of injectable second-line drugs in large population. Because of small number of subjects studied, generalization of the findings to wider population appears difficult. Therefore, current study was conducted to assess the prevalence and management of nephrotoxicity, and ototoxic symptoms, and treatment outcomes of patients treated for MDR-TB with injectable-containing-regimens in two treatment centers in Addis Ababa, Ethiopia.

## Methods

### Study design and setting

The study design is cohort, descriptive and retrospective study conducted using routinely collected clinical and laboratory data, where the data were extracted from patient medical records collected at two MDR-TB treatment centers from January 2010 to December 2015. The study was conducted in St. Peter’s Specialized Hospital and All African Leprosy Rehabilitation and Training Center (ALERT) which are national MDR-TB referral centers in Ethiopia [20].

### Study population and sample description

The study population comprised of all patients who were treated for MDR-TB at St. Peter’s Specialized Hospital and ALERT center during study period and met eligibility criteria. The inclusion criteria was suspected or culture-proven MDR-TB patients without age restriction who have been treated between 1^st^January 2010 and 31 December 2015 and receiving regimens containing any of injectable agents (amikacin, kanamycin, capreomycin, or streptomycin). Patients who were not on injectable-containing regimens and those who have incomplete records were excluded. The total MDR-TB patients treated in St.Peter’s Hospital during study period were about 842, and 600 patients were sampled by simple random sampling technique while 300 patients were included in the study from ALERT center making total sample 900 subjects.

### Data collection and analysis

The subjects were followed-up for 8 months during intensive therapy when they received regimen containing any of injectables for the purpose of recording of nephrotoxicity and ototoxic symptoms associated with injectable treatment, but final treatment outcomes were also recorded after standard duration of treatment. Data were collected by health professionals working in MDR-TB centers from medical records of participants using a pre-tested structured data extraction instrument. The data collectors and supervisors were given training on data extraction procedures, data handling and confidentiality issues. The data collected include sociodemographic characteristics such as age, sex and body weight at baseline and clinical characteristics such as HIV status, antiretroviral therapy (ART), serum creatinine at baseline and follow-up, sputum microscopy, mycobacterial culture results, drug susceptibility test results, types of anti-TB drugs at baseline, other co-morbidities and concomitant medications, description of TB type, the choice of injectable, duration of treatment, average dose of injectable, total dose, reason for stopping injectable treatment, adverse event and its management. Total dose was calculated from the dose, the frequency and the duration of treatment.

For assessment of outcomes of interest, ototoxicity and nephrotoxicity were considered primary outcomes. Ototoxicity symptoms were defined as clinically diagnosed or patient-reported hearing problems; hearing loss, vestibular symptoms (imbalance and visual disturbance) at any point after start of treatment.

Nephrotoxicity was defined as more than 50% increase in serum creatinine from baseline at any time during treatment [9, 22].

Data were statistically analyzed using SPSS software version 22. Descriptive statistics was used to summarize data. Further analysis of factors associated with nephrotoxicity and ototoxicity was not possible due to small observations of primary outcomes compared to large sample of study participants. A *p* value less than 0.05 was considered statistically significant.

### Ethical considerations

Ethical approval of the study protocol was obtained from all concerned bodies including from Institutional Review Board of College of Health Sciences of Addis Ababa University (Ref: 002/2017), Armauer Hanson Research Institute (AHRI)-All African Leprosy Rehabilitation and Training Center (ALERT) Ethics Review Committee (AAERC) (Ref: P005/17 and Emory University Institutional Review Board (Ref: IRB 00093720). The confidentiality of data was maintained via ethical training of data collectors, safe storage of collected data, and finally de-identifying of personal information prior to data analysis.

## Results

### Socio-demographic characteristics

Among 900 patients whose records were reviewed, 21 were excluded due to incomplete records and 879 patient records were included in the final analysis. Overall, 473 (54.2%) of participants were male with age range of 2–75 years. Children accounted for 47 (5.5%) of cases with mean age of 13.6 ± 3.8 years. The mean age of adults was 32 ± 12.6 years and the mean body weight was 48.1 (±10.3) kilograms. Majority of participants (*n* = 405, 43.5%) had a secondary education, while 81 (8.7%) and 47 (5%) of subjects had alcohol drinking and smoking documented, respectively (Table 1).

**Table 1:** Sociodemographic characteristics of study participants at MDR-TB treatment follow-up at St.Peter’s specialized hospital and ALERT center, Addis Ababa, Ethiopia

Sociodemographic characteristics		N (%)
Facility Name	St. Peter’s Hospital	596 (64.0)
	ALERT Center	279 (30.0)
Patient Visit	Inpatient	712 (76.5)
	Outpatient	162 (17.4)
Sex	Male	473 (54.2)
	Female	400 (45.8)
Body weight (kg)	Mean (SD)	48.1 (10.3)
Age (years)	2–17	47 (5.5)
	18–75	819 (94.5)
Pregnancy	Yes	21 (2.3)
Education	No formal education	82 (8.8)
	Primary (from 1 to 6)	157 (16.9)
	Secondary (from 7 to 12)	405 (43.5)
	Tertiary (certificate and above)	198 (21.3)
	Unknown	37 (4.0)
Alcohol	Yes	81 (8.7)
	No	695 (74.7)
	Not documented	103 (11.1)
Alcohol frequency	Once a week	20 (2.1)
	Two times a week	14 (1.5)
	Three times a week	3 (0.3)
	Four times a week	1 (0.1)
	More frequently	16 (1.7)
	Other frequency	13 (1.4)
Smoke	Yes	47 (5.0)
	No	751 (80.7)
	Unknown	81 (8.7)

### Clinical characteristics of study participants

As shown on Table 2, the clinical registration of MDR-TB patients indicates that the majority (*n* = 442, 47.5%) of subjects were registered as *After failure of re-treatment* followed by 213 (22.9%) *After-failure-of-first treatment*, and 124(13.3%) *Relapse cases; New* MDR-TB cases accounted only for 77(8.3%). The majority (*n* = 788, 84.6%) of participants have received TB treatment in the past which suggests that majority of TB patients have acquired resistance against first-line anti-TB drugs. Regarding the diagnosis, 850 (91.3%) were bacteriologically confirmed MDR-TB cases while 27 (2.9%) were clinically diagnosed; and 817 (87.8%) of cases were diagnosed as pulmonary tuberculosis. The sensitivity testing indicated that 242 (26%) were tested by phenotypic drug susceptibility testing while 356 (38.2%) were tested by GeneXpertMTB/RIF and 232 (24.9%) by Line probe assay (LPA).

**Table 2:** Clinical characteristics of study participants during MDR-TB treatment at St.Peter’s Specialized Hospital and ALERT center, Addis Ababa, Ethiopia

Clinical characteristics		N(%)
Case registration	New case	77 (8.3)
	Relapse	124 (13.3)
	After failure of first treatment	213 (22.9)
	After failure of re-treatment	442 (47.5)
	Treatment after being lost to follow- up	4 (0.4)
	Transfer -in	2 (0.2)
	Other	11 (1.2)
TB treatment in the past	Yes	788 (84.6)
	No	63 (6.8)
	Unknown	27 (2.9)
Treatment taken for past TB	First-line drugs	792 (85.1)
	Second- line drugs	25 (2.7)
	Both first and second line drugs	4 (0.4)
Second- line past injectable treatment	Kanamycin	1 (0.1)
	Amikacin	6 (0.6)
	Capreomycin	17 (1.8)
	Streptomycin	33 (3.5)
Anatomical site TB	Pulmonary	817 (87.8)
	Extrapulmonary	59 (6.3)
MTB detection	Bacteriologically confirmed MDR-TB	850 (91.3)
	Clinically diagnosed MDR-TB	27 (2.9)
	NA	2 (0.2)
MTB confirmation	GeneXpert MTB/RIF positive	347 (37.5)
	Culture	234 (25.1)
	Line probe assay	214 (23.0)
	Sputum smear positive	29 (3.1)
Resistance /Sensitivity testing	GeneXpert MTB/RIF	356 (38.2)
	Line probe assay	232 (24.9)
	Phenotypic drug susceptibility testing	242 (26.0)
	NA + other	42 (4.7)
HIV status	HIV positive MDR-TB patients	171 (18.4)
	HIV negative MDR-TB patients	685 (73.6)
	HIV status unknown MDR-TB patients	23 (2.4)
Pattern of resistance	Isoniazid and rifampicin resistance	341 (40.1)
	GeneXpertMTB/RIF rifampicin only resistance	260 (30.6)
	Rifampicin resistance with isoniazid susceptibility	38 (4.5)
	Isoniazid, rifampicin, ethambutol, streptomycin, resistance	132 (15.5)
	Isoniazid, rifampicin and streptomycin resistance	1 (0.1)
	Others	94 (10.1)

The pattern of drug resistance detected among 850 confirmed MDR-TB patients indicated that 341 (40.1%) were both isoniazid and rifampicin resistant, 260 (30.6%) rifampicin only resistant (GeneXpert MTB/RIF positive), 38 (4.5%) rifampicin resistant with isoniazid susceptibility, and 132(15.5%) isoniazid, rifampicin, ethambutol, and streptomycin resistant. Analysis of outcomes of MDR-TB treatment with second-line drugs indicated that treatment was completed in 536 (61.9%), 109 (12.6%) were cured, 3 (0.3%) had treatment failure, 91 (10.5%) died, 52 (6%) were lost to follow-up, and 72 (8.3%) were not further evaluated; 3 patients were moved to XDR-TB registration. The treatment success (cured plus treatment completed) was 645 (74.5%).

### The pattern of longitudinal change in serum creatinine

Nephrotoxicity in study participants was screened using baseline and monthly measurements of serum creatinine and clinical diagnosis based on patient /physician reports. As shown on Table 3, analysis of medical records for serum creatinine data indicated that 808 (91.9%) of patients had serum creatinine records at baseline, and the majority 769(87.5%) of patients had serum creatinine record at first month of intensive therapy, however there was decreasing pattern of number of patients being monitored using serum creatinine for renal toxicity during intensive phase MDR-TB treatment. The mean serum creatinine values increased significantly (p<0.05 for all means) from baseline continuously until the 4th month of treatment, followed by slight decline though values were still significantly above baseline measurements. The mean increase in serum creatinine from baseline was the highest (75%) at 4th month of intensive phase of treatment. The prevalence of nephrotoxicity as indicated by greater than 50% increase of serum creatinine from baseline, increased from first month of treatment (10.79%) to 18.07% at 8th month of treatment. There was an increasing trend of prevalence of nephrotoxicity across follow-up months, however, the number of patients monitored for nephrotoxicity using serum creatinine against treatment months was decreasing. The analysis of medical records indicates that clinically diagnosed nephrotoxicity accounts for 62 (6.7%) cases (Table 4). This prevalence of nephrotoxicity based on subjective patient reports/physician diagnosis as documented on patient medical records is lower than the prevalence of nephrotoxicity based on laboratory analysis of serum creatinine.

**Table 3:** Characterization of study participants based on serum creatinine measurements during intensive phases of MDR-TB treatment at St.Peter’s Specialized Hospital and ALERT center, Addis Ababa, Ethiopia

Intensive phase of treatment (number of subjects on follow-up)	Serum creatinine (mg/dL) (Mean ± SEM) (95% CI)	Mean change of serum creatinine from baseline (%)	N(%) of participants with >50% increase of serum creatinine from baseline
Baseline (*n* = 808)	0.75 ± 0.02 [0.71–0.78]	–	**–**
1st month (*n* = 769)	0.82 ± 0.02 [0.78–0.87]	2.9	83 (10.79)
2nd month (*n* = 717)	0.85 ± 0.02 [0.81–0.89]	20.67	103 (14.36)
3rd month (*n* = 661)	0.84 ± 0.01 [0.81–0.88]	13.3	105 (15.88)
4th month (*n* = 619)	0.86 ± 0.02 [0.81–0.91]	75.6	101 (16.32)
5th month (=582)	0.86 ± 0.02 [0.82–0.91	1.9	90 (15.46)
6th month (*n* = 533)	0.84 ± 0.01 [0.81–0.88]	−33.4	84 (15.75)
7th month (*n* = 482)	0.82 ± 0.01 [0.80–0.85]	−47	76 (15.76)
8th month (*n* = 415)	0.83 + 0.01 [0.81–0.86]	−74.6	75 (18.07)

**Table 4:** Description of injectable regimens, toxicities and discontinuation among participants on MDR-TB treatment follow-up at St.Peter’s Specialized Hospital and ALERT center, Addis Ababa, Ethiopia

Characteristics	N(%)
Type of injectable drug used	
Capreomycin	789 (84.7)
Kanamycin	37 (5.6)
Amikacin	40 (4.3)
Streptomycin	2 (0.2)
Mean (± SD) duration on injectable treatment (months)	8.51 ± 2.3
Mean (± SD) total dose of injectables received(g)	158.5 ± 56.2
Switching of injectable	49 (5.3)
Discontinuation of injectable due to toxicity	34 (3.9)
Hearing disturbance/ototoxicity	8 (0.9)
Nephrotoxicity/clinical renal failure	26 (2.9)
Clinically diagnosed nephrotoxicity	62 (6.7)
Clinically diagnosed ototoxicity	42 (4.8)
Hearing loss	16 (1.8)
Imbalance	7 (0.8)
Visual disturbance	19 (2.2)

### Drug-induced ototoxicity

Clinically diagnosed ototoxicity (ototoxic symptom) was detected in 42 (4.8%) of subjects, of which 16 (1.7%) patients had persistence of ototoxicity symptoms (Table 4). Ototoxicity symptoms were documented as hearing loss, imbalance, and visual disturbance, however audiometric assessments were not practiced in study sites.

### Injectable regiments and management of drug-induced toxicities

The review of use of injectable anti-tuberculosis drugs in study sites indicates that capreomycin 789 (84.7%) was predominantly used during intensive phase of MDR-TB treatment, while kanamycin and amikacin had the same rate of use (Table 4). The analysis of clinical management of ototoxicity and nephrotoxicity during intensive phase treatment of MDR-TB indicates practices like switching between injectables, withholding and discontinuation of injectable administration, decreasing frequency from 6 times a week to two to three times a week, and decreasing standard dose of injectable (Table 5). The injectable in the regimen were switched in 49 (5.3%) participants and injectable therapy was discontinued due to ototoxicity in 8 (0.9%) and clinical nephrotoxicity in 26 (2.9%) cases (Table 4). Therefore, ototoxicity and nephrotoxicity were managed mainly by modification of treatment regimens.

**Table 5:** Clinical management practices of nephrotoxicity and ototoxicity symptoms of injectables in patients on MDR-TB treatment follow-up at St.Peter’s Specialized Hospital and ALERT center, Addis Ababa, Ethiopia

Management practice & injectables involved in toxicity	Nephrotoxicity cases (*N* = 62)	Ototoxicity symptoms (*N* = 42)
Decreased frequency of capreomycin dose to 2x/week, or 3x/week	6	1
Withhold and discontinue capreomycin treatment	28	2
Discontinue, and then taper capreomycin treatment	1	1
Discontinue kanamycin treatment		1
Tapering capreomycin dose from 1000 to 750 mg /day +3x/week	2	1
Stop injection for 15 days	1	
Close follow-up	1	

## Discussion

The present study investigated the prevalence and clinical management of nephrotoxicity and ototoxic symptoms, and treatment outcomes of patients treated for MDR-TB with injectable- based regimens in a large cohort of patients. Aminoglycosides are drugs with a narrow therapeutic index and pharmacokinetic variability that require careful monitoring of serum levels, particularly during their prolonged use in MDR-TB patients, to prevent the occurrence of dose-dependent ototoxicity and nephrotoxicity [23]. In addition, regular audiologic assessments may help in the early detection of hearing impairment, before the damage becomes extensive and irreversible [24].

Analysis of sociodemographics of participants in our study indicates that adults accounted for 95% of MDR-TB cases and pediatric cases were 5%. This proportions are similar to findings in Tanzania [25]. The baseline age (32 ± 12.6) and body weight (48 years) findings in our study were comparable to findings in Namibian study [26]. The majority (84.6%) of participants have received TB treatment in the past which suggest that majority of TB patients have acquired resistance against first-line anti-TB drugs. A case-control study carried out in China indicates that adverse reactions are strong independent predictor for high prevalence of multidrug-resistant tuberculosis among previously treated patients [27]**.**

Analysis of outcomes of MDR-TB treatment with second-line drugs indicated that treatment was completed in 61.9%), while 12.6% were cured and 10.5% died. The treatment success was 74.5% which is greater than findings from systematic review [28] but identical to findings from Tanzania [25]. Our finding is in contrast to Egyptian study [7] where about 88.4 and 2.9% subjects were cured and completed treatment respectively. The mortality finding in our study is comparable to findings from recent systematic review and meta-analysis that indicated pooled death of 12.25% in the course of MDR-TB treatment [29].

Nephrotoxicity in study participants was screened using baseline and monthly measurement of serum creatinine, and clinical diagnosis based on patient reports. The clinical use of nephrotoxic drugs in MDR-TB management may be unavoidable, so understanding mechanisms of their nephrotoxicity, early detection of drug-induced nephrotoxicity and reduction of the incidence of therapeutic side effects are important to avoid the end stage of renal failure [30]). Aminoglycosides induce acute tubular necrosis as primary cause of functional toxicity, and activation of the renin-angiotensin system and the ensuing local vasoconstriction appear to be primarily responsible for the decrease in glomerular filtration. Aminoglycoside-induced nephrotoxicity manifests clinically as nonoliguric renal failure, with a slow rise in serum creatinine and a hypoosmolar urinary output developing after several days of treatment [16]). Other manifestations of nephrotoxicity include urinary casts, proteinuria, and decreased creatinine clearance and increased serum levels of urea. A rise in serum creatinine concentration of more than 0.5 to 1 mg/dL (44 to 88 micromol/L) or a 50% increase in serum creatinine concentration from baseline occurs in 10 to 20% of patients [31, 32].

The diagnosis of drug-induced acute kidney injury (AKI) is currently based on decreased glomerular filtration rate (GFR) and urine output, and increased serum creatinine, blood urea nitrogen and albuminuria. The sensitivity of such nephrotoxicity biomarkers is limited to well-established AKI. Application of selected biomarkers for early diagnosis of drug-induced AKI may inform on progression of AKI and alert clinicians earlier adoption of renoprotective strategies [33]. Creatinine-based methods have also important limitation in older patients [34]. Novel biomarkers, accepted for early detection of drug-induced AKI including kidney injury molecule-1, neutrophil gelatinase-associated lipocalin and N-acetyl-β-d-glucosaminidase, may be useful additions in panels of biomarkers [33].

In the present study, serum creatinine measurements indicates that majority (91.9%) of patients had serum creatinine records at baseline, however, the number of patients being monitored with creatinine sampling was in decreasing order across 8 months of intensive therapy. The decreasing pattern of number of patients being monitored for nephrotoxicity during intensive phase of MDR-TB treatment might be due to patient burdens, lack of continuous supply of laboratory reagents or clinical underestimation of incidence of nephrotoxicity across months of intensive treatment and due to outcomes of treatment such as mortality, lost to follow-up and transfer out.

The mean increase in serum creatinine from baseline was the highest (75%) at 4th month of intensive phase of treatment. The prevalence of nephrotoxicity as indicated by greater than 50% increase of serum creatinine from baseline, increased from first month of treatment (10.79%) to 18.07% at 8th month of treatment. There was an increasing trend of prevalence of nephrotoxicity across follow-up months indicating significant proportion of participants were affected by toxicity of injectable drugs. Previous report [35] has indicated that 25% (21/85) of the patients had a rise in the creatinine blood level from the baseline measurements.

Ototoxicity is the second main adverse effect of aminoglycosides and which, in contrast to nephrotoxicity, is irreversible. Streptomycin is primarily vestibulotoxic, whereas amikacin, and kanamicin are primarily cochleotoxic [36]. Cochlear damage can produce permanent hearing loss, and damage to the vestibular apparatus results in dizziness, ataxia, and/or nystagmus. Aminoglycosides appear to generate free radicals within the inner ear, with subsequent permanent damage to sensory cells and neurons, resulting in permanent hearing loss [36]. Specific conditions can increase the risk of drug-induced ototoxicity, including HIV, malnutrition, aging, high levels of sound, smoking, and alcohol use, prolonged duration of treatment, total accumulated dose,, and concomitant therapy with other therapeutic agents such as loop diuretics and glycopeptides, serious bacterial infections and genetic factors [13, 37].

The review of use of injectable anti-tuberculosis drugs in study sites indicates that capreomycin was predominantly (84.7%) used during intensive phase of MDR-TB treatment, while kanamycin and amikacin had same rate of use. The finding is different from Egyptian study [7] where the utilization preference order of injectables was kanamycin, amikacin and capreomycin. The difference in pattern of use of injectables could be due to supply and price issues of the drugs, and safety experiences. In a study [35] that investigated relative adverse effects of capreomycin and amikacin in MDR-TB patients, ototoxicity was five times more likely for patients started on amikacin than for those treated with capreomycin only, and the likelihood of composite hearing loss was 14 times greater for patients started on amikacin than those treated with capreomycin only indicating that capreomycin had less ototoxic potential compared to aminoglycosides. The analysis of medical records indicates that clinically diagnosed nephrotoxicity accounts for 62 (6.7%) cases while clinically diagnosed ototoxic symptoms were detected in 42 (4.8%) of subjects, of which 16 (1.7%) patients had persistence of such symptoms. This observations are comparable to other studies in the setting which reported prevalence of 8.8% renal insufficiency and 6.0% hearing impairment [21, 38] which reported comparable findings in small cohort of patients. However, ototoxic symptom finding in our study was several times lower than a study from Iran [39] which reported 14.5% auditory toxicity and Pakistan study [40] which reported 21% ototoxicity and a UK study [35] which reported 55% of ototoxicity. The differences might be due to lack of audiometric follow up at current study setting which underestimated our finding while stated studies indicated availability of audiometric assessments. Ototoxicity manifestations were documented as hearing loss, imbalance, and visual disturbance, however audiometric assessments were not practiced in study sites. The prevalence of nephrotoxicity based on subjective patient reports/physician diagnosis as documented on patient medical records is lower than the prevalence of nephrotoxicity based on laboratory analysis of serum creatinine. The differences in clinical and laboratory-based nephrotoxicities might be attributed to clinical under-recognition of nephrotoxicity.

In the present study, 18% of MDR-TB patients were HIV co-infected. Although not shown in our study, some studies indicated the risk of HIV coinfection and ART on aminoglycoside induced-ototoxicity and nephrotoxicity. Individuals with MDR-TB and HIV coinfection had a 22% higher risk of developing aminoglycoside-induced hearing loss than non-HIV-infected individuals [41]. Co-treatment with kanamycin based regimen and ART was associated with an increased risk of renal insufficiency [42]. This observations may indicate the need for injectable-sparing regimens and more frequent monitoring of toxicities for HIV-coinfected individuals.

The analysis of clinical management of nephrotoxicity and ototoxicity symptoms during intensive phase treatment of MDR-TB indicates practices like switching between injectables, withholding and discontinuation of injectable administration, decreasing frequency from 6 times a week to two to three times a week, and decreasing standard dose of injectable. The injectable in the regimen were switched in 49 (5.3%) participants and injectable therapy was discontinued due to ototoxicity in 8 (0.9%) and clinical nephrotoxicity in 26 (2.8%) cases (Table 5). The rate of discontinuation in our finding is less than that of [35] that reported 40% injectable discontinuation due to hearing loss. Therefore, ototoxicity and nephrotoxicity were managed mainly by modification of treatment regimens. Eliminating the need for injectables in MDR-TB treatment regimens has been a high priority [43], accordingly the latest WHO guideline recommends fully oral regimen. If injectables are used, evaluation of potentially otoprotective medications N-acetylcysteine or aspirin, high frequency hearing screening for earlier detection of ototoxicity and therapeutic drug monitoring are essential [44]. Intensive monitoring and management of adverse effects caused by second-line drugs are essential components of drug-resistant TB control programs which can decrease non-adherence and mortality [45].

The present study findings from large study population indicated significant magnitude of toxicity related to second-line injectable anti-tubercular drugs used during intensive phase of MDR-TB, which may be baseline data for clinical management of patients and further study, however, the study has certain limitations due to retrospective design of the study, and lack of audiometric follow up for definitive diagnosis of ototoxicity and the study was descriptive.

## Conclusion

The results indicate that nephrotoxicity and ototoxicity symptoms were significant problems among patients on follow up for MDR-TB treatment and capreomycin was the principal drug used as injectable agent. Based on laboratory criteria (serum creatinine), nephrotoxicity remained significant toxicity throughout intensive phase of treatment indicating the need for close monitoring of patients for successful outcomes and avoidance of long-term renal impairment. Clinically-recognized toxicities were usually managed by modification of treatment regimens.

## Abbreviations

ADR: Adverse drug reaction
FMOH: Federal ministry of health, Ethiopia
MDR/RR-TB: Multi-drug resistant/ Rifampicin resistant tuberculosis
RIF: Rifampicin
WHO: World Health Organization
XDR-TB: Extensively drug resistant tuberculosis
